# The stress hormone corticosterone in a marine top predator reflects short-term changes in food availability

**DOI:** 10.1002/ece3.1438

**Published:** 2015-02-26

**Authors:** Robert T Barrett, Kjell E Erikstad, Hanno Sandvik, Mari Myksvoll, Susi Jenni-Eiermann, Ditte L Kristensen, Truls Moum, Tone K Reiertsen, Frode Vikebø

**Affiliations:** 1 Department of Natural Sciences, Tromsø University MuseumPO Box 6050 Langnes, Tromsø, NO-9037, Norway; 2 Norwegian Institute for Nature Research, FRAM – High North Research Centre for Climate and the EnvironmentTromsø, NO-9296, Norway; 3 Centre for Biodiversity Dynamics, Department of Biology, Norwegian University of Science and TechnologyTrondheim, NO-7049, Norway; 4 Institute of Marine ResearchPO Box 1870 Nordnes, Bergen, NO-5817, Norway; 5 Swiss Ornithological InstituteSempach, CH-6204, Switzerland; 6 Marine Genomics Research Group, Faculty of Biosciences and Aquaculture, University of NordlandBodø, NO-8049, Norway

**Keywords:** Common guillemot, CORT, food availability, seabird, *Uria aalge*

## Abstract

In many seabird studies, single annual proxies of prey abundance have been used to explain variability in breeding performance, but much more important is probably the timing of prey availability relative to the breeding season when energy demand is at a maximum. Until now, intraseasonal variation in prey availability has been difficult to quantify in seabirds. Using a state-of-the-art ocean drift model of larval cod *Gadus morhua*, an important constituent of the diet of common guillemots *Uria aalge* in the southwestern Barents Sea, we were able to show clear, short-term correlations between food availability and measurements of the stress hormone corticosterone (CORT) in parental guillemots over a 3-year period (2009–2011). The model allowed the extraction of abundance and size of cod larvae with very high spatial (4 km) and temporal resolutions (1 day) and showed that cod larvae from adjacent northern spawning grounds in Norway were always available near the guillemot breeding colony while those from more distant southerly spawning grounds were less frequent, but larger. The latter arrived in waves whose magnitude and timing, and thus overlap with the guillemot breeding season, varied between years. CORT levels in adult guillemots were lower in birds caught after a week with high frequencies of southern cod larvae. This pattern was restricted to the two years (2009 and 2010) in which southern larvae arrived before the end of the guillemot breeding season. Any such pattern was masked in 2011 by already exceptionally high numbers of cod larvae in the region throughout chick-rearing period. The findings suggest that CORT levels in breeding birds increase when the arrival of southern sizable larvae does not match the period of peak energy requirements during breeding.

## Introduction

Predicting the effects of climate variability on and through the different trophic levels is a major challenge, and one that increases in complexity up the food chain. Within the marine ecosystem, effects of climate on life-history traits have been documented across many species and populations (reviewed in Ottersen et al. 2010), including seabirds (e.g., Frederiksen et al. 2006; Sandvik and Erikstad 2008; Sandvik et al. 2012), often as a result of perturbations in food supply (reviewed in Furness 2007; Bustnes et al. 2013; Erikstad et al. 2013). In many studies, single annual proxies of prey abundance have been used to explain variability in seabird breeding performance (reviewed in Furness 2007), but much more important is probably the timing of the prey relative to the breeding season (reviewed in Durant et al. 2005) when energy demand is at a maximum (Markones et al. 2010). This is referred to as the *match–mismatch hypothesis* and was first proposed for marine systems where the interannual variation in fish recruitment was suggested to depend on the timing of the production of their food (Hjort 1914; Cushing 1990). However, as pointed out by Durant et al. (2005), tests of the hypothesis have often only addressed the temporal variation in food availability and ignored the variance in food abundance that could compensate for small temporal mismatches. Seabird breeding seasons are timed to coincide with peak food supply, and seasonal variations in the latter act as a major selective force determining the breeding season (Lack 1968; Ashmole 1971). As such, any trophic mismatch may decouple breeding phenology from food availability and is thus an important determinant of fitness in seabirds (Burthe et al. 2012). In predictable environments, animals can adapt in anticipation of change and thereby reduce stress as indicated by levels of circulating glucocorticosteroids (Wingfield 2003), but global warming can likely upset this predictability, and one of the most consistent consequences is the disruption of reproductive function (Wingfield and Sapolsky 2003).

**Figure 1:**
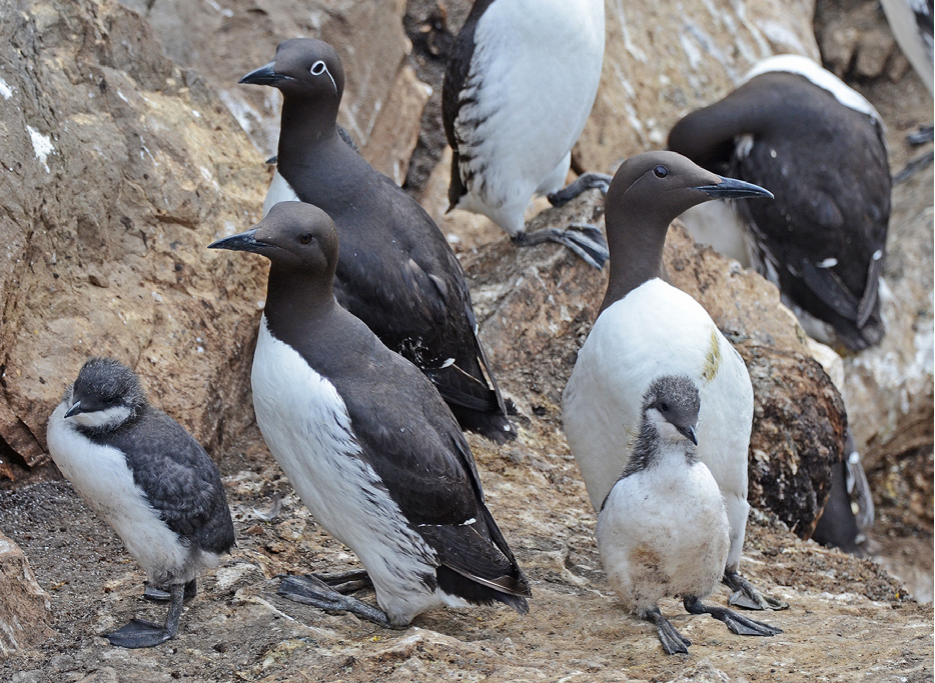
Adult common guillemots with 2- to 3-week-old chicks shortly before they leave the nest ledge. Hornøya, North Norway, July 2013. Photo: Rob Barrett.

Seabird survival, breeding success, diet, and foraging effort have often been used to infer prey availability and the effects of environmental factors thereon at a seasonal time scale, but it has proved extremely difficult to determine the effects of within-season changes in prey abundance on these top predators (reviewed by Benowitz-Fredericks et al. 2008). Whereas reproductive parameters may not be a reliable measure of prey availability (Benowitz-Fredericks et al. 2008), recent studies have shown that one practical alternative is to measure endocrine changes. In particular, levels of stress hormones are known to correlate with food supply (and survival) over short and long intervals (Wingfield and Kitaysky 2002; Benowitz-Fredericks et al. 2008; Doody et al. 2008; Satterthwaite et al. 2012 and refs. therein), and measurements of the glucocorticoid hormone corticosterone (CORT, an important regulator of energy metabolism) have been used to infer changes in the ecosystem (e.g., Benowitz-Fredericks et al. 2008; Satterthwaite et al. 2012; Kouwenberg et al. 2013). Such studies have, for example, revealed a direct, negative correlation between levels of CORT in the bloodstream and food abundance among common guillemots *Uria aalge* (Fig.1) (Kitaysky et al. 2007) and barn swallows *Hirundo rustica* (Jenni-Eiermann et al. 2008).

**Figure 2:**
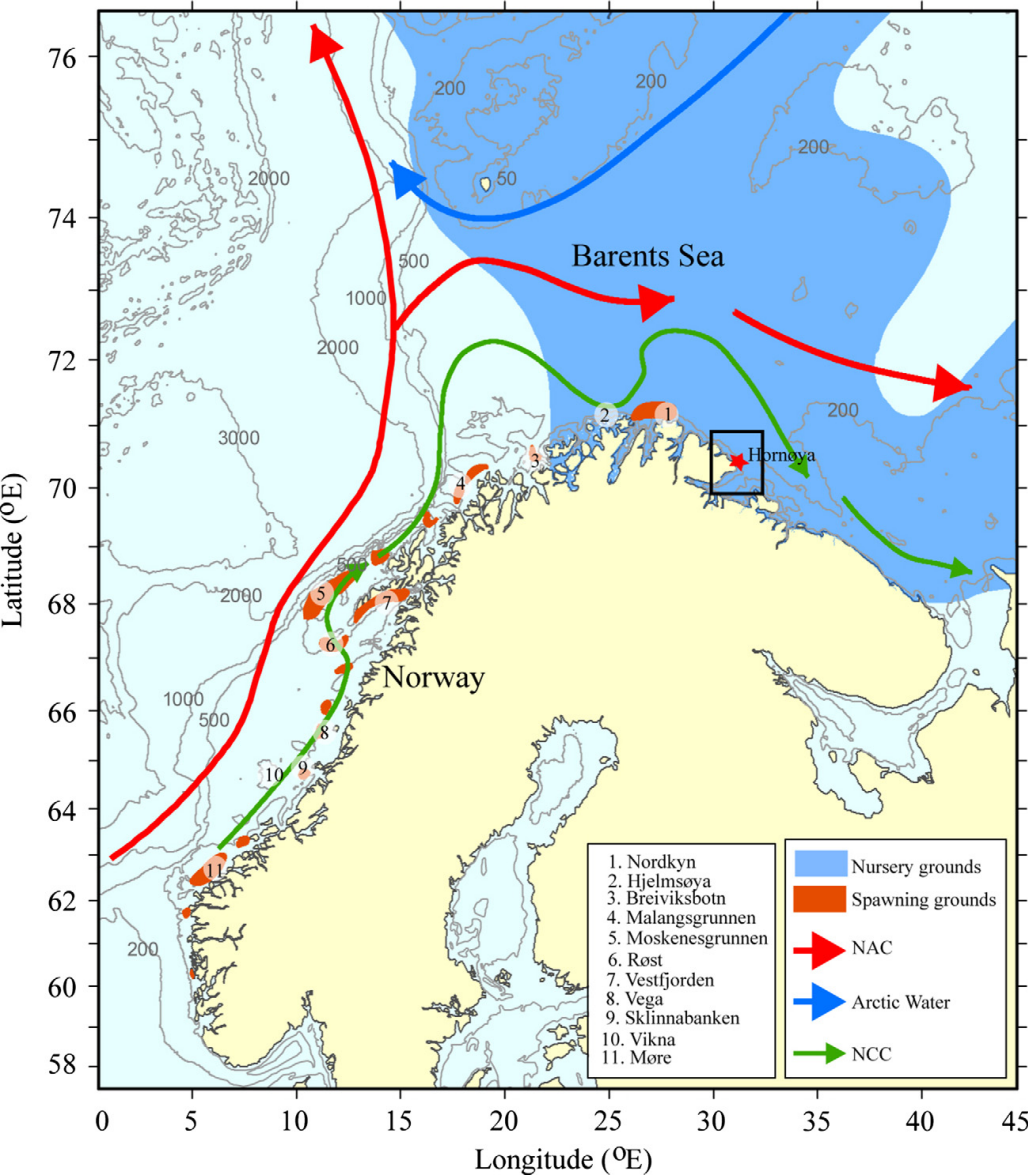
The study area including the most important current features, the Norwegian Atlantic Current (NAC) and the Norwegian Coastal Current (NCC), Northeast Arctic cod spawning areas numbered from north to south and the common guillemot colony at Hornøya (red star) centered in the approximate foraging area of chick-feeding adults (black box). Spawning areas 1–2 are “northern,” and 4–11 are the “southern” spawning areas.

The Barents Sea is a highly productive shelf ocean where the local variability in production is highly dependent on the transport of water from the Norwegian Sea, either by the nearshore Norwegian Coastal Current (NCC) or by the offshore Norwegian Atlantic Current (NAC) (Loeng 1991) (Fig.2). Of the biota, the Northeastern Arctic (NEA) stock of cod *Gadus morhua* dominates the Barents Sea ecosystem through its great abundance, wide distribution, long migrations, and omnivorous feeding habits (Yaragina et al. 2011). The spawning grounds are widely distributed along the Norwegian coast, and spawning occurs in early spring (March–April) along the coast between SW Norway and Finnmark (Fig.2). Spawning occurs pelagically, and the early life stages (eggs and larvae) are transported northwards along the coast into the Barents Sea (Dalpadado et al. 2012) by the NCC and NAC, a transport that takes them past many seabird colonies within the foraging range of the breeding adults. The amount of juvenile cod available to the seabirds at each colony depends on the numbers of larvae, their growth during their drift northwards and their survival rates, and may be a key factor influencing the breeding performance of predators such as seabirds.

Determining the relationships between fish stock dynamics, including recruitment rates, and the environment has long proved difficult. However, using a biophysical model and a three-dimensional ocean circulation model, Svendsen et al. (2007) were able to show that the mean flow of Atlantic water through the entrance to the Barents Sea in October–December prior to the year of spawning and the modeled monthly mean April primary production in the entire Barents Sea could explain 70% of the variability of 3-year-old cod recruits. Recently, an ocean circulation model with high temporal and spatial resolution (Lien et al. 2013a) was coupled to a cod larval model developed by Ådlandsvik and Sundby (1994) and based on known spawning grounds distributed along the Norwegian coast, the model provides daily drift patterns for cod larvae (Vikebø et al. 2005; Kristiansen et al. 2009). The model has successfully proven to reproduce the spatial distribution of 0-group cod in the Barents Sea (Vikebø et al. 2011) and was used to show the influence of the availability of cod larvae on the size of common guillemot chicks (Myksvoll et al. 2013).

The common guillemot is a very common, circumpolar, boreal, and low-Arctic seabird species (Gaston and Jones 1998). The adults are long-lived and produce a maximum of one chick a year that is fed single individuals of small, energy-rich pelagic fish (Fig.1). The chicks have an intermediate fledging pattern whereby they leave the breeding site when 15–21 days old and 15–35% of adult mass, long before they can fly. Recent studies have shown that adult North Norwegian common guillemots were at times much dependent on 0-group NEA cod as prey during the breeding season. Although chicks were fed on energy-rich Atlantic herring *Clupea harengus*, capelin *Mallotus villosus* or sand eels *Ammodytes* sp., most of the adult diet consisted of the two youngest year classes of Gadidae, probably NEA cod and haddock (*Melanogrammus aeglefinus*) (Bugge et al. 2011). Of the two species, 0-group cod is believed to be the most favoured and is an important determinant of the annual survival and fitness of adults during the breeding season (Barrett and Erikstad 2013; Erikstad et al. 2013; Myksvoll et al. 2013).

Although successfully demonstrated in the terrestrial ecosystem (Jenni-Eiermann et al. 2008), it has until now been impossible to relate seasonal changes in baseline CORT directly to independent day-to-day measures of food availability in the marine ecosystem. The aim of this study was therefore to relate levels of baseline CORT in common guillemots breeding in Northern Norway with concurrent and independent real-time measures of food availability within the foraging range of the population. By doing this, we could, for the first time, study in detail the within-season timing of guillemot breeding in relation to both the arrival and the changes in abundance of an important food item in the waters around the colony and how the timing of breeding may affect the CORT level of adult guillemots. The larvae drift model mainly estimates the interannual variations in drift patterns and as a control for the modeled variations, we used independent annual measures of the abundance of 0-group cod in the Barents Sea in August (i.e., after the guillemot breeding season) (Eriksen et al. 2009; SJØMIL 2014). We predicted that (1) levels of baseline CORT in adult breeding common guillemots during the peak period of energy demand would decrease as daily amounts of cod larvae available in the waters around the breeding colony increased and/or (2) the growth and survival of the guillemots’ offspring would increase in inverse relationship with adult CORT levels.

## Material and Methods

### Field methods

The study was carried out in June and July 2009, 2010, and 2011 at Hornøya (70°22′N, 31°09′E, Fig.2), a 0.5 km^2^ island in NE Norway that then hosted a colony of approximately 10,000 breeding pairs of common guillemot (R. Barrett, unpubl. data). Thirty-six pairs (with their chick) were sampled in 2009, 39 in 2010, and 32 in 2011, resulting in 107 family-seasons. Because 24 pairs were sampled in one season only, 25 in two and 11 in three seasons, the sample consisted of 60 independent families. The families were sampled in plots within a 100 m^2^ area at the top of a large subcolony of approximately 250 pairs (see Kristensen et al. (2014) for details). All birds were permanently marked individually using color rings. Family membership was determined by observations of nest site fidelity and which adults incubated an egg and tended the chick. The exact hatching dates of all chicks were recorded, and adult birds were captured around the hatching date (*N *=* *204 observations, 60 independent families) using a 4–6 m extendable noose pole, and recaptured 12 days (±4 days) later (*N *=* *182 observations, 58 independent families). Blood samples (0.3 mL) were taken for DNA-based sexing (first capture day) and plasma CORT analysis (both capture days). The samples were taken within 3 min of capture to achieve baseline values of CORT (Romero and Reed 2005; Doody et al. 2008). The blood was centrifuged within 6 h, and plasma was stored frozen at −18°C until CORT analysis. Chicks were also captured within 3 ± 2 days of hatching (*N *=* *108 observations, 60 independent families) and recaptured when 15 ± 1 days old (*N *=* *94 observations, 56 independent families). Chicks were weighed (±5 g), and their tarsus lengths (±0.1 mm) were measured. Additionally, a blood sample (25 *μ*L) was taken at first capture for DNA-based sexing. Chick growth was defined as the average daily mass gain between the two captures. Chick survival was defined as survival from hatching to day 15 posthatch.

### Laboratory methods

As adult guillemots cannot be phenotypically sexed, a DNA-based sexing technique (Griffiths et al. 1998) was used. DNA was extracted with the Nexttec™ Genomic DNA Isolation Kit for Tissue and Cells, version 3.1 (Biotechnologie GmBH, Hilgertshausen, Germany). Regions of the sex-linked CHD-Z, and CHD-W genes (*c*hromobox-*h*elicase-*D*NA-binding gene) were PCR amplified using P2/P8 primers (Griffiths et al. 1998), and the PCR products analyzed by electrophoresis on 2% agarose gels. Females were identified by two distinguishable bands in the electrophorese gel analysis in contrast to a single band for males. With the possible exception of the ratites (ostriches, etc.), this test has been proved to be robust for avian species, including the common guillemot (Griffiths et al. 1998; Birkhead et al. 2001).

Plasma CORT concentration was measured using an enzyme immunoassay (EIA, Munro and Stabenfeldt 1984; Munro and Lasley 1988). Plasma pools from birds with two different CORT concentrations were included as internal controls on each plate. The detection limit of the assay was 1.21 ng/mL. If the concentration was below detection threshold, the value of the lowest detectable concentration was assigned (11 samples). Intra-assay variation ranged from 7.7 to 10.1%, and interassay variation from 7.2 to 10.4% depending on the internal controls. Full details are given in Kristensen et al. (2013). CORT measurements were log_10_-transformed prior to analysis.

### Larval drift model

Based on Sundby and Nakken (2008), we selected the 11 most important spawning grounds for NEA cod distributed along the Norwegian coast, as shown in Fig.2, starting from the north: 1. Nordkyn, 2. Hjelmsøya, 3. Breivikbotn, 4. Malangsgrunnen, 5. Moskenesgrunnen, 6. Røst, 7. Vestfjorden, 8. Vega, 9. Sklinnabanken, 10. Vikna, and 11. Møre. Of these, the most important are those around the Lofoten archipelago (5–7), although the spawning activity is known to shift northwards during warm periods (Sundby and Nakken 2008). In the model, 300 particles (representing cod eggs) are released every third day from each of the 11 spawning grounds throughout March and April (in total 69,300 eggs), the main spawning season, and develop into larvae after a fixed egg stage duration of three weeks. Because the physical environment has high variability, both in time and space, it is important to use a constant number of particles in the model each year, even though the actual spawning stock biomass of NEA cod may vary through the period. In this way, we can estimate the interannual variations in drift patterns. The eggs and larvae drift northwards in the NAC and NCC toward their nursery grounds in the Barents Sea. The ocean climate variables were extracted from a model archive with 4 km horizontal resolution covering the Nordic Seas and the Barents Sea produced with the ocean circulation model ROMS (Regional Ocean Modeling System) (Lien et al. 2013a,b). The physical oceanographic conditions determine both the vertical distribution of the offspring as well as the horizontal dispersal, as described by Myksvoll et al. (2013). When running the model on an annual basis, an accumulation of particles within a 100 × 100 km box (i.e., the approximate foraging range of common guillemots around Hornøya; Fig.2) was determined on a daily basis during the breeding season of common guillemots. Yearly variations in actual numbers of larvae were proxied using trawl data indices from annual ecosystem cruises made in August (Eriksen et al. 2009; SJØMIL 2014).

### Statistical analyses

As explanatory variables, we considered abundance and mass of larvae from the eleven spawning grounds in addition to sea surface temperature (SST) within the box around Hornøya. Baseline CORT levels mirror the stress birds have experienced during the days or week preceding measurement (Kitaysky et al. 2010). We therefore aggregated larval abundance for a period of 7 days up to and including the day of CORT measurement. SST was averaged over the same 7-d period.

Many of the variables exhibited high levels of correlation (Supplementary Table S1), which prohibited their inclusion in the same analysis. Whereas the sign of the correlation between larvae from the different spawning grounds varied between years, the correlation between the two northernmost areas was always highly positive, as was the correlation between the eight southernmost areas (Supplementary Table S1). We therefore defined two new variables, “southern larvae,” which was the sum of larvae from spawning grounds 4–11, and “northern larvae,” the sum of larvae from spawning grounds 1–2 (Fig.2). Larval biomass was highly correlated with larval abundance (*r* ≥ 0.96; Supplementary Table S1), and because they were very similar to the results obtained using abundance (cf. Supplementary Table S2), we do not present results from analyses including mass.

Analyses were performed using the statistical packages SAS (SAS Institute Inc 2008) and R (R Development Core Team 2011). The statistical dependence of multiple observations from the same families was taken into account by means of mixed-effects models, using family (breeding pair) as a random factor (Bates et al. 2011). Models were compared using Akaike's information criterion (AIC) and presented using its difference (ΔAIC) from the best model's AIC. The model with the lowest AIC that at the same time had an AIC at least 2 units smaller than any nested simpler models, was considered the best model. Estimates and their corresponding standard errors (SE) are presented as “mean ± SE”. Probabilities of covariates are based on likelihood ratio tests between a model with and an otherwise identical model without the focal covariate.

## Results

**Figure 3:**
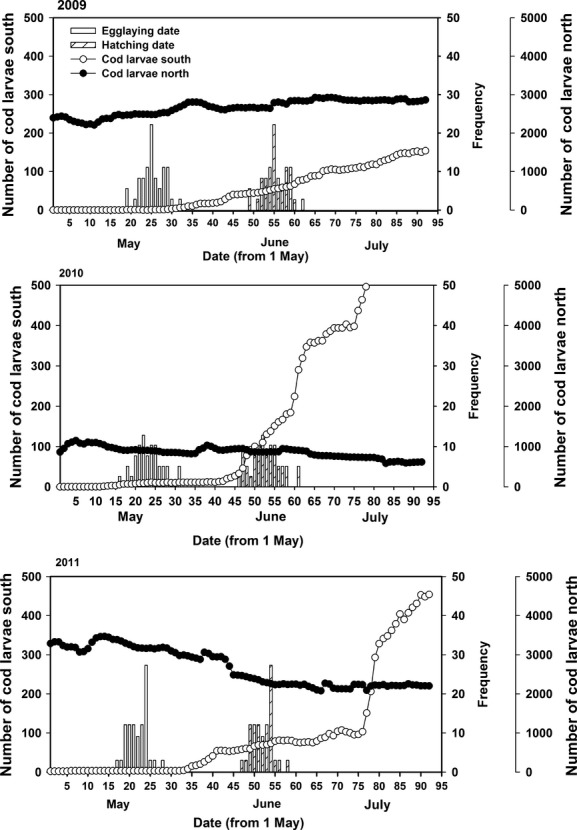
Modeled numbers of cod larvae from southern (4–11) and northern (1–2) spawning areas within the approximate foraging area of adult common guillemots breeding on Hornøya, NE Norway, in relation to egg-laying and hatching dates in 2009, 2010, and 2011. (See Fig.2 for spawning areas and foraging range). (Frequency = no. of eggs laid or hatched d^−1^).

According to the model, the abundance of cod larvae around Hornøya varied between years, depending on the area of origin (southern vs. northern spawning grounds), and throughout the guillemot breeding season (Fig.3). Northern larvae arrived at Hornøya in near constant streams and at much higher frequencies than those from the south throughout all three breeding seasons. In contrast, the amount of southern larvae differed greatly between years, as did the timing of their arrival. In 2009, the abundance of southern larvae was low during the entire breeding season, although it did increase slightly in the latter part of the season. In 2010, a sudden wave of southern larvae arrived during the hatching period. In 2011, a similar wave of larvae arrived ca. 25 days after the hatching period. The abundance of cod larvae from southern and northern spawning areas was strongly correlated, although the sign varied between years (2009, *r* = +0.94; 2010, *r* = −0.83; 2011, *r* = −0.90). The abundance of southern cod larvae was positively correlated with SST (2009, *r* = 0.84; 2010, *r* = 0.87; 2011, *r* = 0.93; see Table S1 for other correlations).

The CORT levels in breeding common guillemots differed between years (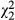 = 21.67, *P *=* *0.00002) and between the sexes (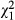 = 4.98, *P *=* *0.026), and there was a significant interaction between year and time within the season (i.e., at hatching vs. at 15 days posthatch; 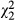 = 7.72, *P *=* *0.021; see Table1). The remaining main effect (time) and interactions (sex × time, sex × year, sex × time × year) were not significant (all *χ*^2^ < 4, all *P *>* *0.1). Several pronounced patterns emerged when the availability of cod larvae was added to the models. Abundance of larvae from the southern spawning grounds turned out as a crucial parameter (Table2), because both its main effects (CORT levels decrease with increasing larval numbers) and its interaction with year were highly significant (neighboring models are shown in Supplementary Table S2).

**Table 1:** Overview of baseline CORT levels (ng ml^−1^) of common guillemots breeding at Hornøya, NE Norway. Values are presented as means ± standard errors (sample size). The sample is subdivided by year (2009, 2010, 2011), sex (female vs. male), and time (“early” = at hatching, “late” = 15 days after hatching)

	2009	2010	2011
Males			
Early	8.38 ± 0.04 (35)	6.02 ± 0.05 (38)	5.88 ± 0.05 (29)
Late	6.82 ± 0.05 (33)	6.53 ± 0.05 (32)	5.38 ± 0.06 (26)
Females			
Early	8.49 ± 0.05 (33)	5.01 ± 0.06 (37)	3.65 ± 0.06 (32)
Late	6.60 ± 0.07 (32)	6.56 ± 0.06 (33)	4.78 ± 0.06 (26)

**Table 2:** Parameter and model statistics of the best model explaining variation in log_10_-transformed CORT levels of common guillemots breeding at Hornøya, NE Norway across all years. The parameters selected are time (hatching vs. 2 weeks later), year (2009, 2010, 2011), and larvae/S (abundance of cod larvae from southern spawning grounds, cumulated over the seven days prior to measurement of corticosterone levels). The model is a mixed-effects model including breeding pair as a random variable (which accounted for 18% of the variance, *N *=* *55 breeding pairs)

Parameter	Estimate ± SE	*t*	*χ* ^2^	*P*
Time	0.246 ± 0.064	3.87		0.00013
Year	−0.539 ± 0.120	−4.49		<10^−5^
Larvae/S	−1.269 ± 0.263	−4.82		<10^−5^
Year × larvae/S	1.140 ± 0.239	4.77		<10^−5^
Model (*N *=* *344)			67.01	<10^−11^

**Figure 4:**
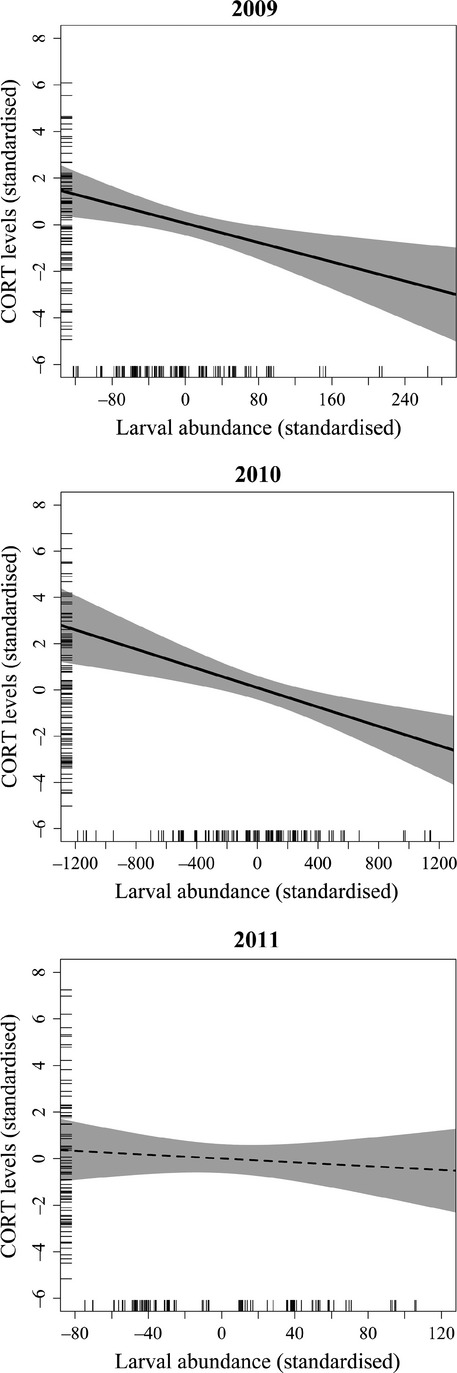
Baseline CORT levels (log_10_-transformed) in adult common guillemots breeding at Hornøya, NE Norway in relation to abundance of cod larvae from southern spawning grounds (regression line and 95% confidence intervals) within the guillemots’ foraging area. The plots correct for variation between the sexes and within the season (early vs. late), such that the axes are standardized to a mean of zero. Note that the scales of the *x*-axes differ between years. Ticks indicate the distribution of the variables.

Because of the strong effect of year, the variation in CORT levels was in the next step analyzed separately for different years (Table3, Fig.4). In 2009 and 2010, CORT levels decreased in guillemots that were caught after a week with high abundance of cod larvae (Fig.4). The corresponding tendency in 2011 was not significant. Patterns were similar in both sexes (Table3). CORT levels tended to be higher during late than during early measurements (Table3).

**Table 3:** Models explaining variation in log_10_-transformed baseline CORT levels of common guillemots breeding at Hornøya, NE Norway, in three different years. Further subdivisions by sex (female vs. male) and time (hatching vs. 2 weeks later) are also given. Separate models were fitted for each subdivision of the sample. Only parameters that were at least marginally significant (*P *<* *0.1) were retained. Estimates are given as mean ± SE (sample size). Asterisks indicate significance levels (0.1 > *P*^+^ ≥ 0.05 > P^*^ ≥ 0.01 > P^*^^*^ ≥ 0.001 > P^*^^*^^*^). No interactions were supported. “Larvae/N” refers to cod larval abundance from northern spawning grounds, cumulated over the seven days prior to measurement of CORT levels (see Tables1 and 2 for explanations of the remaining variables)

Group	2009		2010		2011	
	Parameter	Estimate	Parameter	Estimate	Parameter	Estimate
Overall	Larvae/S	−1.24 ± 0.37^*^^*^ (121)	Larvae/S	−0.24 ± 0.06^*^^*^^*^ (126)	–	(97)
	Time	+0.23 ± 0.11^*^ (121)	Time	+0.43 ± 0.10^*^^*^^*^ (126)		
Females	Larvae/S	−0.61 ± 0.27^*^ (59)	Larvae/S	−0.26 ± 0.08^*^^*^ (64)	Larvae/S	−1.69 ± 0.99^+^ (49)
			Time	+0.47 ± 0.14^*^^*^ (64)	Time	+0.25 ± 0.13^+^ (49)
Males	Larvae/S	−1.55 ± 0.49^*^^*^ (62)	Larvae/S	−0.21 ± 0.09^*^ (62)	–	(48)
	Time	+0.35 ± 0.15^*^ (62)	Time	+0.39 ± 0.15^*^ (62)		
Early	Larvae/S	−0.91 ± 0.41^*^ (60)	Larvae/S	−0.24 ± 0.09^*^^*^ (64)	Sex	+0.22 ± 0.08^*^^*^ (50)
Late	Larvae/S	−1.71 ± 0.65^*^ (61)	Larvae/S	−0.24 ± 0.08^*^^*^ (62)	Larvae/S	−2.37 ± 1.20^+^ (47)
					Larvae/N	−0.80 ± 0.32^*^ (47)

The correlation between southern and northern larvae made it difficult to disentangle the respective effects statistically. However, when entering larvae from both areas as covariates, northern larvae reached significance late in 2011 only; this effect was only apparent when the amount of southern larvae was included in the same model (Table3). Models of CORT variations across years and seasons were poorer when northern larvae were used instead of southern larvae (the best model incorporating northern larvae had a ΔAIC of 33; cf. Supplementary Table S2). Although the abundance of southern cod larvae was positively correlated with sea surface temperature, temperature was too poor a proxy to explain variations in CORT levels (the best model incorporating SST had a ΔAIC of 30).

Chick growth and late chick body mass (corrected for age) varied between years (Table4) and were highest in 2010, the year with the highest abundance of southern larvae. However, intraseasonal variation in late chick body mass was not related to their parents’ CORT levels or to the amount of southern larvae (all *R*^2^ < 0.1 < *P*). Chick growth was negatively related to the amount of southern larvae late in the 2009 breeding season (*R*^2^ = 0.21, *P *=* *0.010), but not in the other years or with the parents’ CORT levels (all *R*^2^ < 0.1 < *P*). Survival of chicks was relatively high (86%, Table4), stable, and unrelated to abundance of southern larvae and CORT levels in all years (all |*z*| < 1.1, all *P *>* *0.2).

**Table 4:** Mean hatching dates and mean sizes ± 1 SE (*N*) of common guillemot chicks and mass of cod larvae at Hornøya, NE Norway. Chick measurements are on the nest site 15 days posthatch. Chick growth is mass gained per day between hatching and day 15. Larval measurements are based on a larval drift model and are provided for day 15 posthatch. Biomass of larvae is the product of larval size and the abundance index. All models are mixed-effects models including year as main effect and breeding pair as random effect

Parameter	2009	2010	2011	*χ* ^2^	*P*
Hatching date (day in June)	24.3 ± 0.5 (36)	21.5 ± 0.6 (39)	21.1 ± 0.6 (33)	25.55	<10^−5^
Chick survival	0.89 (36)	0.87 (39)	0.82 (33)	0.70	0.71
Chick body mass (g)	192.7 ± 5.1 (32)	240.2 ± 6.5 (35)	236.5 ± 7.1 (27)	48.24	<10^−10^
Chick growth (g day^−1^)	6.6 ± 0.6 (32)	10.6 ± 0.9 (34)	11.0 ± 0.9 (27)	25.76	<10^−5^
Chick tarsus length (mm)	38.7 ± 0.3 (32)	39.9 ± 0.4 (34)	40.1 ± 0.5 (26)	9.51	0.0086
Mass of southern larvae (mg)	107.5	88.5	82.2		
Mass of northern larvae (mg)	43.0	33.4	29.3		
Biomass of southern larvae	11,288	33,542	7312		
Biomass of northern larvae	144,432	36,840	92,004		

## Discussion

Through the unique resolution of the ocean drift model providing a daily proxy of larval numbers within the foraging range of adult seabirds, this study was able, for the first time, to document short-term effects of changes in food availability on CORT levels in breeding seabirds. The drift model revealed considerable day-to-day variation in the numbers of cod larvae in the guillemot foraging area around Hornøya to which baseline CORT levels in breeding common guillemots responded, as predicted, through a decrease with increasing numbers of prey in two of the three years of the study (2009 and 2010, Fig.4). In 2011, there was no such relationship due to small changes in numbers of larvae during the breeding season. In 2009, there was a slow and very gradual influx of southern larvae into the waters around the colony starting during the incubation period, whereas in 2010, there was a near perfect match between food availability and chick rearing with southern larvae starting to arrive already during the hatching period and increasing rapidly in numbers such that parents of chicks could access an ever increasing abundance of food. As such, southern larvae explained much of the CORT variation that season. Although a similar wave of larvae arrived at Hornøya in 2011, it was too late in that it did not arrive until well after the second CORT blood samples were taken and even after most of the chicks had left the nest site (pers. obs.).

Although the modeled frequencies and overall biomass of larvae from the northern spawning grounds were much higher than those from the south every year, especially in 2009 and 2011 (Table4, Fig.3), there was very little variation in the former during the guillemot breeding season, thus excluding them as a factor explaining the short-term variations in CORT. From their different response to the two sources of larvae, one can also infer that the guillemots selected for cod larvae from the southern spawning grounds, despite their lower modeled frequency. This is likely due to the greater size (Table4) and hence higher energetic value (Ball et al. 2007) of the southern larvae that, when arriving at Hornøya, have drifted northwards for a longer time and in warmer water (hence enhancing their growth) than their northern counterparts (Myksvoll et al. 2013).

Many other factors may influence baseline CORT levels in breeding seabirds, for example, sex (as found in this study), life-history strategy, population status, extreme weather, year, breeding stage, sex allocation in offspring, and/or breeding experience (Romero et al. 2000; Lanctot et al. 2003; Kitaysky et al. 2010; Kristensen et al. 2013; Schultner et al. 2013), and thus potentially interfere with our results. Our analyses were, however, based on changes in CORT levels rather than on absolute values, thus ruling out many of these confounding factors. It was, furthermore, limited to pairs that had already survived the first stages of the breeding season with no egg loss and thus better “socially established” (Kitaysky et al. 2007).

Comparisons of CORT levels found in this study with those reported in Doody et al. (2008), who investigated whether a time of 3 min between capture and blood sampling resulted in true baseline CORT levels in common guillemots, reassure us that our sampling procedure did not result in elevated values. Values of 4–7 ng mL^−1^ (Table1) are below the minimum levels recorded in Doody et al.'s (2008) experiment. They also correspond to the baseline levels of food-related unstressed guillemots in Kitaysky et al. (2007). As such, there is no evidence of a handling-induced elevation of baseline levels in this study.

Early guillemot studies have suggested that changes in baseline CORT levels can be a result of changing food conditions, but they were based on either indirect measurements of the food base (sampling of regurgitations, responses to supplementary feeding, etc.) or on single measures of peak food availability (Doody et al. 2008). At shorter time scales, Kitaysky et al. (2007) found a negative relationship between baseline CORT to independent food abundance measurements taken every 2 weeks, whereas Doody et al. (2008) sampled at a ca. 1 week interval before and after peak food availability. Our study was more finely tuned and was able to relate CORT levels directly to changes in daily proxies of food availability. The short-term responses documented also corroborate earlier studies that underline baseline CORT as a better proxy of food availability than measurements of the more traditional breeding parameters (e.g., chick growth and breeding success) (Kitaysky et al. 2010; Satterthwaite et al. 2012; Smout et al. 2013). However, like in the study by Lanctot et al. (2003), inconsistencies did arise between measures of productivity and CORT levels.

**Figure 5:**
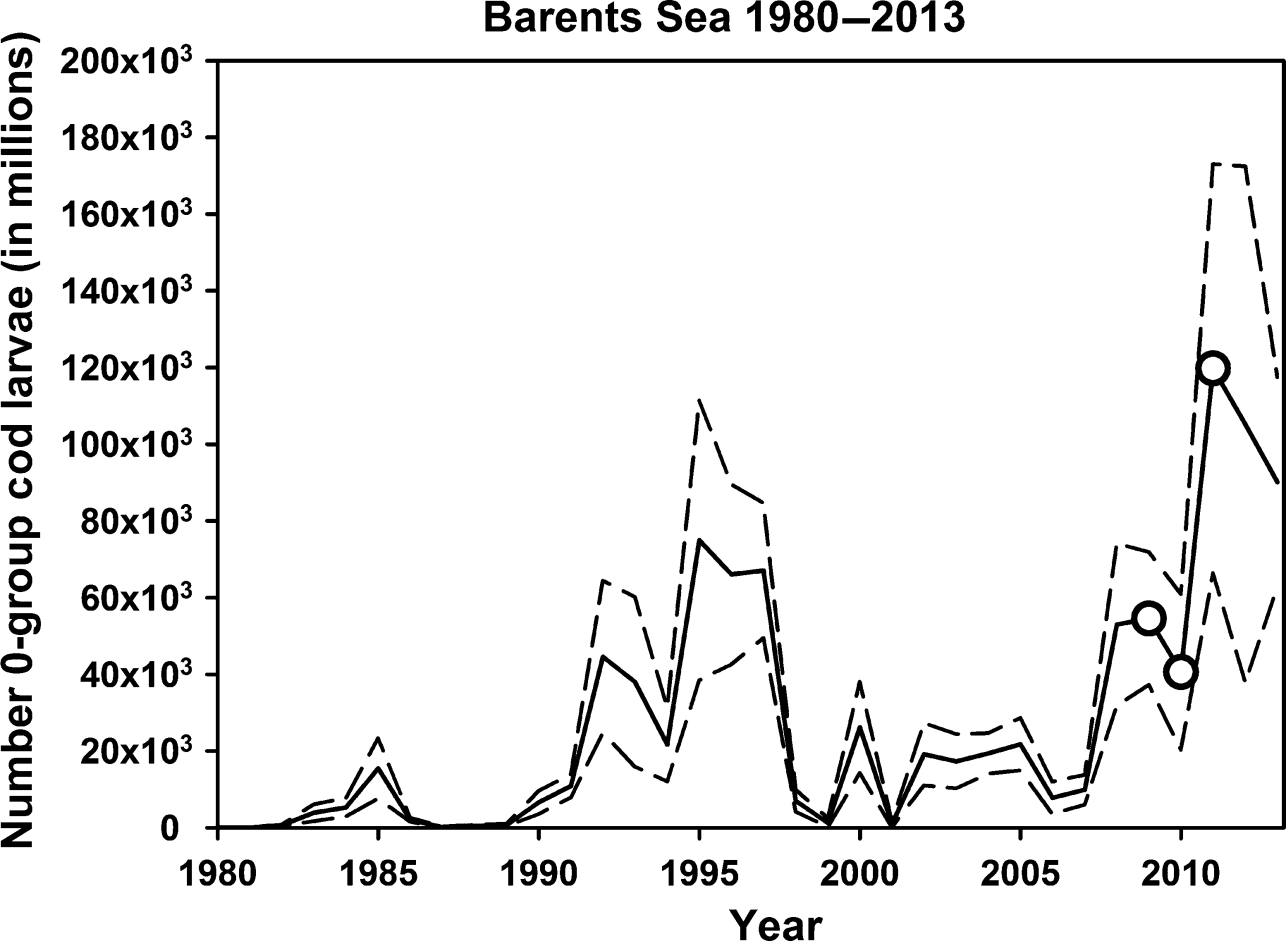
Abundance indices (in millions, with 95% confidence limits) of 0-group cod in the Barents Sea in August 1980–2013. Data from SJØMIL (2014).

Whereas the intraseasonal variation in CORT levels fulfilled our prediction that more larvae would result in lower CORT levels (at least in the two years when there was sufficient variation in larval abundance), the pattern was not as clear on an interseasonal basis. Comparing the two-first years, CORT levels were lower in 2010 than in 2009, possibly due to the highest availability of southern cod larvae during the chick-rearing period in 2010 (Table4). In 2011, on the other hand, CORT levels were even lower despite the modeled low frequencies of larvae within the foraging area and the clear mismatch between the arrival of southern larvae and the guillemot chick-rearing period. However, while the larval drift models capture the spatiotemporal variation in drift patterns, they are silent about the absolute number of cod spawning products. Based on empirical estimates of the latter (SJØMIL 2014), 2011 was an exceptionally good year. These numbers of cod larvae in the Barents Sea are estimated during an annual cruise in August, and while lacking the resolution of the larval drift model, they capture the total abundance of 0-group cod. The estimate for 2011 was the highest since measurements began in 1980 and more than twice the numbers recorded in 2009 and 2010 (Fig.5, Table5). This suggests a continuous presence of an easily accessible food supply for the adults in 2011. Moreover, as much as 75% of the cod stock spawn in the Lofoten area (southern area), a fraction that is rather stable between years (Sjølingstad 2007). This thus contributes to continually high numbers of large larvae present around Hornøya this year, thereby reducing the guillemots’ workload during foraging bouts and hence CORT levels throughout the chick-rearing period.

**Table 5:** Abundance indices (in millions, with 95% confidence limits) of 0-group cod in the Barents Sea in August of the 3 years of this study (2009–2011). Data from SJØMIL (2014)

Year	Abundance indices	95% CI
2009	54,579	37,311–71,846
2010	40,635	20,307–60,962
2011	119,736	66,423–173,048

Chick survival, chick growth, and late chick mass were not related to the interannual variation in the frequency of southern larvae within the foraging range of the adult birds or in their parents’ CORT levels. At first sight, this seems counterintuitive, but may have been due to the adults never having to increase their effort to a threshold above which their offspring were affected. In all three years, the adults seem to have been able to find enough food for themselves and their chicks, thereby buffering reproduction from variability in the availability of cod. This suggests that despite differences in apparent 0-group cod availability from the southern spawning grounds, there was enough alternative prey near the colony, or that any further increase in consumption rates and hence growth is limited by the adults’ handling and/or the adults’ and chicks’ digestion rates (Jeschke et al. 2007; Smout et al. 2013). However, before the arrival of the southern larvae, parents presumably had to work harder to obtain enough food, which may explain the elevated CORT levels during these periods. As such, baseline CORT levels seem to be a good proxy of short-term changes in food availability. Due to their near consistent response to daily food proxies, they also provide a useful medium through which to investigate the match–mismatch hypothesis.

## Data Accessibility

The data underlying our analyses are available from the Dryad Digital Repository (doi:10.5061/dryad.cb5h8).

## Supporting Information

**Table S1.** Correlation matrix of explanatory variables. **Table S2.** Models explaining variation in baseline CORT levels of common guillemots breeding at Hornøya, NE Norway across three years, sorted by decreasing fit (i.e. increasing ΔAIC).
