# Supplementary material for: The stress hormone corticosterone in a marine top predator reflects short-term changes in food availability
Source: Ecol Evol. 2015 Feb 26;5(6):1306–17. doi: 10.1002/ece3.1438 (PMC4377273; doi:10.1002/ece3.1438)
Supplement: Supplementary file 1 [file ece30005-1306-sd1.docx]

ECE-2014-10-0483.R1 Supplementary tables

| Table S1. Correlation matrix of explanatory variables. Variables are numbers of cod larvae from eleven spawning areas (Fig. 2), the total number of cod larvae from areas 4–11 (South), the cumulative mass of cod larvae from areas 4–11 (mass), and sea surface temperature (SST) around Hornøya, NE Norway. The matrix provides the *lowest* yearly correlations obtained for each pair of variables in 2009 to 2011 (e.g. the correlation between areas 1 and 2 was 0.89 in one year and higher in the two other years). Correlations that did not have the same sign in all three years are indicated as "±". Values that were unavailable for two of the three years are indicated as missing ("."). | | | | | | | | | | | | | | | |
| --- | --- | --- | --- | --- | --- | --- | --- | --- | --- | --- | --- | --- | --- | --- | --- |
|  | Mass |  | Cod larvae from spawning area | | | | | | | | | | |  | SST |
|  |  | 1 | | 2 | 3 | 4 | 5 | 6 | 7 | 8 | 9 | 10 | 11 | |  |
| South | 0.96 | ± | | ± | 0.63 | 0.95 | 0.92 | 0.83 | 0.39 | . | 0.92 | 0.83 | 0.85 | | 0.83 |
| Mass |  | ± | | ± | 0.43 | 0.92 | 0.82 | 0.91 | 0.56 | . | 0.89 | 0.77 | 0.91 | | 0.86 |
| Area 1 |  |  | | 0.89 | ± | ± | ± | –0.73 | –0.47 | . | ± | ± | ± | | ± |
| Area 2 |  |  | |  | ± | ± | ± | –0.34 | –0.18 | . | ± | ± | ± | | ± |
| Area 3 |  |  | |  |  | 0.65 | 0.63 | 0.49 | ± | . | 0.62 | 0.23 | 0.23 | | 0.44 |
| Area 4 |  |  | |  |  |  | 0.76 | 0.94 | 0.41 | . | 0.81 | 0.84 | 0.71 | | 0.80 |
| Area 5 |  |  | |  |  |  |  | 0.57 | 0.38 | . | 0.95 | 0.81 | 0.83 | | 0.81 |
| Area 6 |  |  | |  |  |  |  |  | 0.29 | . | 0.69 | . | . | | 0.79 |
| Area 7 |  |  | |  |  |  |  |  |  | . | 0.34 | . | . | | 0.54 |
| Area 8 |  |  | |  |  |  |  |  |  |  | . | . | . | | . |
| Area 9 |  |  | |  |  |  |  |  |  |  |  | 0.60 | 0.82 | | 0.85 |
| Area 10 |  |  | |  |  |  |  |  |  |  |  |  | 0.50 | | 0.57 |
| Area 11 |  |  | |  |  |  |  |  |  |  |  |  |  | | 0.71 |

| Table S2. Models explaining variation in baseline CORT levels of common guillemots breeding at Hornøya, NE Norway across three years, sorted by decreasing fit (i.e. increasing ΔAIC). Variables considered were year (three levels, viz. 2009, 2010, 2011), time (hatching vs. two weeks later), abundance of southern cod larvae (continuous) and sex (female vs. male). Only the best model (boldface) and simplifications thereof are shown, in addition to the best model incorporating southern larval biomass (mass), sea surface temperature (SST) and northern cod larvae (larvae/N). Probabilities are based on comparisons between each model and the corresponding model lacking the last term. All models are mixed-effects models including breeding pair as a random variable. | | | | | | |
| --- | --- | --- | --- | --- | --- | --- |
| Model | Number of parameters | | | ΔAIC | χ^2^ | P |
| **year + time + larvae/S + year×larvae/S** | | 9 | 0.00 | | 23.56 | < 10^–5^ |
| year + larvae/S + year×larvae/S | | 8 | 12.95 | | 8.76 | 0.013 |
| year + larvae/S | | 6 | 17.71 | | 21.63 | < 10^–5^ |
| year + time + larvae/S | | 7 | 19.57 | | 21.73 | < 10^–5^ |
| year + time + sex + mass + year×mass + time×mass | | 11 | 19.90 | | 4.21 | 0.040 |
| larvae/S | | 4 | 27.97 | | 29.04 | < 10^–7^ |
| year + time + sex + year×time + mass | | 10 | 29.53 | | 5.14 | 0.023 |
| time + larvae/S | | 5 | 29.79 | | 29.13 | < 10^–7^ |
| year + sex + larvae/N | | 7 | 32.57 | | 4.02 | 0.045 |
| year | | 5 | 37.34 | | 21.67 | 0.000020 |
| year + time | | 6 | 39.30 | | 0.05 | 0.83 |
| sex | | 4 | 52.04 | | 4.98 | 0.026 |
| NULL | | 3 | 55.01 | | — | — |
| time | | 4 | 56.92 | | 0.10 | 0.76 |
